# The Application of Informatics in Delineating the Proof of Concept for Creating Knowledge of the Value Added by Interprofessional Practice and Education

**DOI:** 10.3390/healthcare3041158

**Published:** 2015-11-12

**Authors:** Frank Cerra, James Pacala, Barbara F. Brandt, May Nawal Lutfiyya

**Affiliations:** 1 National Center for Interprofessional Practice and Education, University of Minnesota, Minneapolis, MN 55455, USA; E-Mails: cerra001@umn.edu (F.C.); brandt@umn.edu (B.F.B.); 2 Department of Family Medicine and Community Health, University of Minnesota, Minneapolis, MN 55455, USA; E-Mail: pacal001@umn.edu

**Keywords:** national center for interprofessional practice and education, informatics and the nexus innovations network, informatics and the NCDR, national center data repository

## Abstract

The resurgence of interest in the promise of interprofessional education and collaborative practice (IPECP) to positively impact health outcomes, requires the collection of appropriate data that can be analyzed and from which information and knowledge linking IPECP interventions to improved health outcomes might be produced and reported to stakeholders such as health systems, policy makers and regulators, payers, and accreditation agencies. To generate such knowledge the National Center for Interprofessional Practice and Education at the University of Minnesota has developed three strategies, the first two of which are: (1) creating an IPECP research agenda, and (2) a national Nexus Innovation Network (NIN) of intervention projects that are generating data that are being input and housed in a National Center Data Repository (NCDR). In this paper, the informatics platform supporting the work of these first two strategies is presented as the third interconnected strategy for knowledge generation. The proof of concept for the informatics strategy is developed in this paper by describing: data input from the NIN into the NCDR, the linking and merging of those data to produce analyzable data files that incorporate institutional and individual level data, and the production of meaningful analyses to create and provide relevant information and knowledge. This paper is organized around the concepts of data, information and knowledge—the three conceptual foundations of informatics.

## 1. Introduction

In the fall of 2012, the National Center for Interprofessional Practice and Education (hereafter the National Center) [1] was established at the University of Minnesota as a partial response to a reaffirmation of the promise of interprofessional health care teams to make significant and meaningful contributions to improving the health outcomes of both individual patients and population groups as well as re-designed health care delivery in the United States (US) [2,3]. With the Triple Aim [4] as a galvanizing force, and results from the success of teams in other business and military sectors, care provided by interprofessional healthcare teams has again been hypothesized as having the potential to not only impact but to also improve patient healthcare quality and the health of populations resulting in a reduction of the per capita cost of care.

Interprofessional education and collaborative practice (IPECP) have engaged the imaginations of health professions educators, clinicians and healthcare providers, and health-oriented researchers for decades. While there has been some struggle settling on shared definitions of IPECP, presently, the most widely accepted definitions of each of these terms are:

Interprofessional education (IPE) “*occurs when two or more professions learn about, from, and with each other to enable effective collaboration and (to) improve health outcomes.*” [5].

Interprofessional Collaborative Practice (CP) *“…happens when multiple health-related workers from different professional backgrounds work together with patients, families, care givers and communities to deliver the highest quality of care.*” [6].

IPECP has been an area of inquiry for more than 40 years [7,8,9,10,11,12]. Many review papers have been published [8,9,10,11,12] about the field offering critical assessments, drawing conclusions about the state of the science as well as making suggestions for future directions. Educators, health professionals, healthcare researchers and policy makers alike have, since the mid-1970s, acknowledged that IPECP has the potential to play a leading role in improving healthcare delivery and health outcomes [8,10]. Given the plethora of readily available reviews written about the state of the IPECP field, we have chosen to focus this paper on the informatics dimension of the National Center.

A decade ago, D’Amour and Oandasan [7] conceptualized interprofessional education and collaborative practice (IPECP) as existing in an intersected space. The National Center refers to this intersected space as the nexus [8] and based on work completed by National Center staff [8] has joined others [9,10,11,12] in recognizing and calling for the necessity of objective, scientifically sound, and rigorously generated evidence assessing and ascertaining if interprofessional education (IPE) and/or collaborative practice (CP) impacts health outcomes such as better patient care quality, population health improvement, and health care cost reduction.

In this regard, the National Center has planned and begun implementing three deliberate strategic approaches: (1) the development and articulation of a research agenda for IPECP within the current US health reform context [13]; (2) the creation of a Nexus Innovation Network (NIN) of interventions to conduct intervention research testing well-designed IPECP models with outcomes clearly linked to those of the triple aim [14]; and (3) developing a National Center Data Repository (hereafter NCDR) creating a sustainable informatics platform using the recognized building blocks of data, information and knowledge [15,16,17,18,19].

The first two strategic endeavors—developing a research agenda and creating and supporting a nexus incubator network for intervention research—have been described elsewhere [13,14]. The significance of the informatics infrastructure supporting the National Center’s research agenda and the intervention research carried out by the NIN should not be underestimated. Developing this informatics strategy as part of a complex and intertwined approach is a sound methodology to address, among other things, some of the major issues that have plagued the field of IPECP [8,9,10,11,12]; most particularly, the need to generate data from multiple well-designed interventions that can contribute to the creation of generalizable knowledge regarding the possible impact of IPE on CP and CP on health-related outcomes [8,12,13,14]. No IPECP-related endeavor that we know of has actually synthesized a research agenda, intervention research network, and informatics platform. This infrastructure is essential in order to move the IPECP field forward.

In this paper, we describe and provide proof of concept for the third strategic endeavor of developing a sustainable informatics platform for the work of the National Center. The domains for this proof of concept are summarized in Table 1 and include data entered into the NCDR by NIN intervention sites, a functioning relational database, and the production of knowledge from analyses.

**Table 1:** Nexus innovation network-national center data repository proof of concept components.

Domains of Proof	Components of Domains	Demonstrable Functions
1. Data Entered into NCDR Relational Database	1.0 National Innovation Network IPECP Interventions (NIN)	1.0 Completion of NCDR Surveys
		1.1 Validation of NCDR Survey inputs
2. Relational Database Function	2.0 Completed individual surveys	2.0 Mapping of database survey fields to analysis plan
	2.1 Data imported from each NIN project	2.1 Production of data analysis results from individual and multiple NCDR Surveys
3. Analysis Reports from Relational Database	3.0 Anecdotal reports	3.0 Anecdotal stories, success factors, lessons learned
	3.1 Qualitative information reports	3.1 Qualitative knowledge from the evaluation of education and collaborative practice processes
	3.2 Quantitative evidence reports	3.2 Quantitative knowledge from NIN project outputs and outcomes

**Figure 1:**
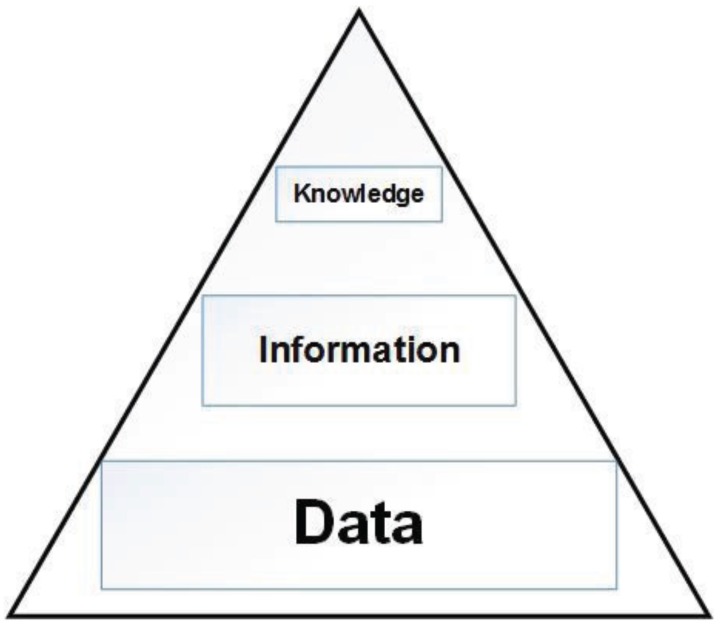
Informatics Triangle.

The NCDR informatics platform is discussed under the headings of data, information and knowledge—the foundational concepts of informatics [15,16,17,18,19]. In the Discussion Section, the fourth informatics foundational concept—wisdom—will be referenced in regard to the work undertaken by the National Center. As a starting point of reference, informatics encompasses the principles and processes through which data are transformed into information with information in turn transformed into actionable knowledge for problem solving and assessment of effect [18,19]. Often the concepts of data, information, and knowledge are depicted as a pyramid (Figure 1), with knowledge as the highest and smallest tier, data as the largest and lowest tier, and information constituting the middle tier of the pyramid. According to Rowley [20], there will always be more data than information, and more information than knowledge.

## 2. Data

Data are discrete observations providing the raw material for information. In scientific research, data are generated or collected through multiple methods (e.g., quantitative or qualitative or mixed) relying on numerous research study designs (e.g., cross-sectional, quasi-experimental or pre/post study designs, randomized control trials, or participant observational). How well and carefully data are collected or generated is crucial to ensure that the largest essential tier of the informatics pyramid is sound and above reproach. Data are, for the most part, unprocessed but must be collected, generated and stored in such a manner that they can be processed or analyzed.

To begin addressing the challenge of the lack of data to test or ascertain if IPECP impacts health outcomes and to assess its ability to provide the return on investment of interprofessional care delivery models, the National Center has developed and is currently populating a relational database—the NCDR. The NCDR is housed in the Academic Health Center Information Systems at the University of Minnesota to store de-identified, secondary data from electronic health records (EHRs) and survey instruments generated in each of the research intervention projects of the NIN [13,14]. The architecture of the database was designed and developed by the informatics group of the University of Minnesota, in cooperation with the Center for Translational Science Institute. It underwent end-to-end testing for input and output functionality, and was repeatedly tested, by both internal and external experts, for safety, security, compliance with privacy regulations, and ability to receive data from multiple users. Since the data stored in the NCDR for analysis is secondary, de-identified data, the University of Minnesota’s Institutional Review Board (IRB) has determined that human subject’s oversight is not necessary for research using these data and carried out by the National Center. Nevertheless, human subject’s oversight is necessary at the intervention project site level and as such local IRB approval must be obtained before data are collected and entered into the NCDR.

**Table 2:** National center data repository surveys.

Survey	Respondents	Questions	Response Type	Time to Complete
**Demographics**	All participants	6 questions creating a personal profile	Multiple choice	<5 min
**Education Survey**	Project lead with input from associated educational unit(s)	24 questions about the IPE program (one survey per unique facility or site)	Multiple choice with open text	15–20 min after 1–2 h of gathering information from multiple relevant sources
**Costs Survey**	Project lead with consultation from all relevant others.	25 questions related to general finances (one survey per unique facility or site)	Multiple choice with open text	30 min after 1–2 h of gathering information from multiple relevant sources
**Network User Survey**	All clinical and educational participants in the intervention (e.g., clinicians, faculty)	32 questions related to IPE and CP at the intervention clinical performance site	Multiple choice with open text	20–30 min after 1–2 h of gathering information from multiple relevant sources
**Student User Survey**	All students participating in the intervention	16 questions related to related to IPE and CP at the intervention clinical performance site	Multiple choice with open text	20–30 min after 1–2 h of gathering information from multiple relevant sources
**Intervention Specific Survey**	Project lead with consultation from all relevant others.	80 questions related to the care processes of the specific project	Multiple choice with open text	45 min after 2–3 h gathering information from multiple relevant sources
**Intervention Outcome Survey**	Project lead with consultation from all relevant others.	Delineation of all outcomes being measured as well as how and when they are being measured	Open text questions	20 min
**Critical Incidents Survey**	Any clinical or educational participant in the intervention (e.g., clinicians, faculty)	5 questions asking the who, what, where, when, how of the incident and your subsequent actions and completed only when a “critical incident” occurs	Open text questions	5–20 min depending on the extent of the issue

NCDR survey instruments were developed with consultation from an internal interdisciplinary expert group, a national/international interdisciplinary expert group, and the National Center’s NCDR Advisory Council. At present, the NCDR is comprised of data collected from multiple surveys. These surveys are described in Table 2. Among the data being collected and stored in the NCDR are:

- demographic;
- network user data;
- student user data;
- education-specific indicators;
- health cost outcome indicators;
- IPE and/or CP costs incurred for the project intervention;
- ecological and environmental factors;
- critical incidents;
- intervention specific data;
- project specific outcomes; and
- process of care descriptors.

The data entered into the NCDR is reviewed and discussed through an ongoing dialog between the National Center and each intervention research project using a variety of means and tools, including conference calls, webinars, network face-to-face meetings, and site visits by National Center staff to projects sites. The latter is particularly needed to verify project cost input data.

The recruitment approach for NIN projects, adopted by the National Center, is at once thoughtful and aggressive. The number of intervention research projects change almost daily as new projects are on boarded. Information about the NIN can be found on the National Center’s website [1] where frequent updates are made. As more intervention research projects are developed and recruited into the NIN, the volume of data collected and stored in the NCDR will increase substantially, and will eventually be large enough to be considered big data. These data provide the basic building blocks for sound knowledge generation regarding IPE and/or CP and examining possible links to triple aim-derived health-related outcomes.

The data from completed surveys populate the NCDR relational database. As such, the data collected by each separate survey (e.g., education survey or network user survey) can be linked together to create a data file for analysis. Linking data using an identification variable shared by each survey allows for the maximization of data. For instance, a data file linking education specific indicators that are collected by institutional level respondents may be linked (in a one to many fashion) to the network user survey data allowing for the analysis of data joined from both surveys. Moreover, the surveys collect both quantitative or quantifiable data as well as qualitative (from open text responses) data. Integrating these different types of data is essential to the knowledge construction process.

## 3. Information

Information is processed data and is needed to create knowledge. Essentially, information is data that has been given some meaning by relational connection and context (e.g., univariate description or bivariate analysis). In and of itself, the meaning may or may not be useful to address a problem. Information is always contextualized data. For example: the number “7” residing in a database is datum. However, once that number is coupled or paired with the variable “A1c” it begins to take on meaning. The number 7 would take on a different meaning if it were the value or score derived from the completion of a “Patient Health Questionnaire” (PHQ-9). The number 7 as datum needs a context or relationship to other pieces of data in order to become meaningful or information. “*Information answers questions that begin with basic words such as who, what, where, when, and how many.*” [16].

Some information is generated from NIN projects using an ethnographic method including observations at site visits coupled with informant interviews. Among the information collected in this manner are: factors the project informants consider essential for their success or failure, and lessons learned during the process of design, implementation and evaluation of interventions. Examples of lessons learned are displayed in Table 3. Some of this information is also captured in the various NCDR surveys. All of the information is important for contextualized understanding and interpreting each project’s experiences in examining IPECP interventions linked to health and healthcare outcomes. Information collected during site visits, once written up, can be housed in the NCDR.

**Table 3:** Preliminary lessons and success factors from the nexus innovation network for IPECP linked to outcome improvement.

Lessons	Factors
The redesign of the process of care is about changing a culture	To include prevention, population health and engagement of people and communities in the redesign and new process of care
	To move from volume to value; fee for service to more global payment systems; new models of care; more care delivered in homes and communities
	To move from teaching to learning, including experiential and on-the-job learning
	To include evaluation and assessment systems of people, teams and programs for their influence and effects on improving health and education outcomes
	To use information and evidence in real-time regarding new models of care and outcome-based decision-making
Moving educational and delivery systems requires a compelling vision and case statement	Information and evidence from the literature and the field are essential
	Return on investment is a common need for all stakeholders
	Leaders, champions and early adopters and early wins are essential
	Learners at all levels, including educators, patients, administrators, regulators and policy makers need to see and understand the value added in the redesign of the process of care
	Partnerships across sectors within and between institutions is essential
The IPECP effort needs to be appropriately resourced	IPECP needs to be part of the strategic plan, goals and direction
	IPECP needs to be positioned high in the organization with operational alignment across the various sectors of the organization
	IPECP needs to be part of the institutional budgeting and accountability processes
Leadership is essential	The effort needs to be visibly championed, from C-suite to learning and clinical settings
	There needs to be an environment where risk is OK to take and manage
	Frequent, transparent communications greatly contribute to success
	Accountability in data collection and reporting is essential
	Education and training in data production methods is essential

Qualitative data are generated from questions on surveys through open text responses. For instance, the narrative generated from the question, “*how is the patient and family considered in care plans?*” This could be a follow-up question to one asking: Are the patient and family considered in care plans, with a yes/no multiple-choice answer. The narrative(s) generated from respondent answers can be thematically analyzed leading to information and eventually new knowledge.

**Figure 2:**
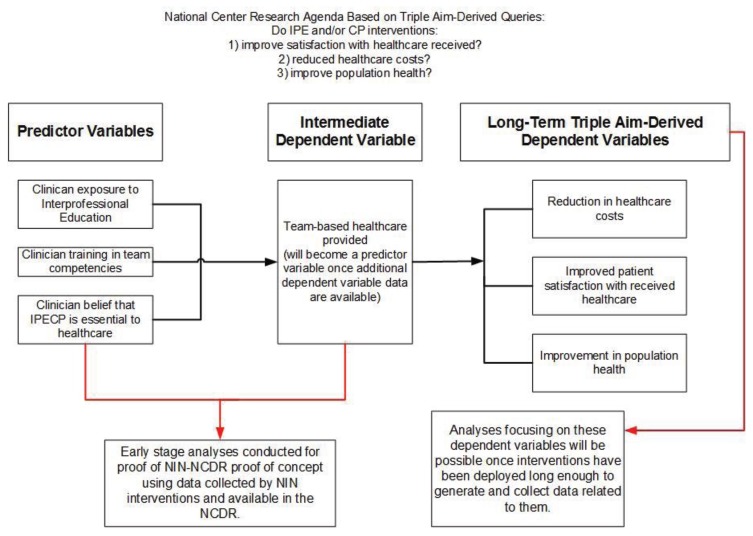
Proof of concept analyses in context of long-term analyses plan.

With the data being collected by the NCDR surveys it is possible to create descriptive information in response to such questions as:

- What are the IPECP interventions being implemented at nexus sites?
- Who are the IPECP interventions team members at each nexus site?
  Life Cycle Inventory
- What are the outcome metrics for IPECP interventions being implemented?
- How many of the IPECP interventions have outcomes that are linked to at least one of the triple aim outcomes?

In addition to these descriptive queries, the analysis processes can provide information and evidence addressing the core queries the National Center has established as part of its research agenda [13]. How does IPECP:

1. Improve the triple aim outcomes on an individual and population level?
2. Result in sustainable and adaptive infrastructure that supports the triple aim outcomes of both education and practice?
3. Identify ecological factors essential for achieving triple aim outcomes?
4. Identify factors essential for systematic and adaptive infrastructure in the transformation of the process of care and education?
5. Identify changes needed in policy, accreditation, credentialing and licensing for health care provision and education?

Control data are also being collected for each team intervention project. Data for each team intervention project is collected longitudinally until the intervention is completed and the health and education outcomes are clarified. In some projects, the data will continue to be collected for an indefinite time after the team intervention is completed. This might be necessary to assess the sustainability of change in the process of care and its potential for transportability. Sufficient data in the NCDR will facilitate analyses focused on the core queries potentially creating actionable knowledge. Until such a time as complete triple aim-derived outcome data are available (generated, collected and entered into the NCDR), intermediate level dependent variables can and will be identified for preliminary analyses. This process is displayed in Figure 2.

## 4. Knowledge

While the concept of knowledge has proven to be more difficult to precisely define in the field of informatics, for our purposes knowledge is derived from the modeled analysis of information leading to the discovery or identification of patterns and relationships between types of information. Furthermore, knowledge is the application of data and information to answer how questions as well as why questions [16,18]. One accepted definition, not entirely different from the one we have adopted, is that knowledge is the synthesis of information or the analysis of information identifying and formalizing relationships [21].

Descriptive analysis yields information whereas knowledge results from hypothesis testing or model development of some sort. As an example, the purpose of using multivariate logistic regression as an analytic technique (this being one technique among many possible techniques) is to allow a researcher to isolate the relationship between an exposure or predictor variable (e.g., receipt of interprofessional education or training in team competencies) and an outcome or dependent variable (e.g., improvement in quality of health care or provision of team-based care) from the effects of one or more other variables (e.g., covariates or possible confounders). As an analytic technique logistic regression allows one to answer the question, “*how do identified ecological variables affect the probability of (or odds of) cost gains, after accounting for—or unconfounded by—or independent of—health care provider type, intervention team composition, or receipt of interprofessional education?*” This analysis process—accounting for covariates or confounders—is also called adjustment.

In keeping with the definition of knowledge as that of identifying and formalizing relationships—examining a well-designed IPECP intervention’s impact on defined outcomes through the multivariate analysis of relevant collected data, will yield knowledge about the effect size different exposure or predictor variables have independent of or separately from one another regarding an outcome or dependent variable. The effect size specifies the contribution of an exposure variable to the outcome being studied (e.g., the effect of interprofessional health care teams on improvement in population health). Tested models, then provide knowledge that can be acted upon in a multiplicity of ways (e.g., creating new or modifying existing IPECP interventions for implementation in relevant health care practice or health professions education settings).

The National Center has mapped the variables or data fields on all of the NCDR surveys to contribute analyses to each of the core queries of the research agenda [13]. This mapping constitutes an analysis and underscores the connection between the data being collected and the research questions being asked. At present there is sufficient data to provide proof of concept regarding the utility of the NCDR. Still awaiting collection and analysis is project outcome data at a sufficient volume for meaningful knowledge creation. Such analyses will provide the subject of future publications.

## 5. Proof of Concept

Proof of concept efforts typically rely on small *n* studies to demonstrate feasibility. In this case, that entails demonstrating the successful linking of data collected from different NCDR surveys and performing meaningful univariate, bivariate and multivariate analyses in order to answer questions about the impact of IPE on CP and eventually on triple aim derived health outcomes. Also possible and essential to the proof of concept for the NCDR is the thematic analysis of qualitative data collected from open text questions. The analyses we present here are for demonstration purposes only. We are presenting analyses of NCDR data, collected from multiple NIN intervention research projects, in order to establish that such can be done successfully. We have chosen to use state of the art/science biostatistics for this analysis.

Presently, intervention project specific outcome data from NIN research projects are being collected but the volume remains insufficient to include as part of the proof of concept of the NCDR. Although this is a small but significant limitation, it is nevertheless possible to link institutional level data collected using the educational survey with individual level data collected from the network user survey (based on a one to many relationship).

Eighteen institutions completed the education survey and 211 individuals (clinicians and educators in intervention performance sites) completed the network user survey. Linking these two databases yielded 203 cases (eight persons completing the network user survey did not have a corresponding institution completing the education survey). Table 4 displays an example of descriptive results using 10 variables collected from the education survey. Univariate analysis revealed that 84.2% of the network users worked at educational institutes with a designated IPE center or office. Further, the results yielded that for those same network users, 97.0% worked at educational institutions where each major clinical site had an IPE champion or lead, 84.7% had a formal method of engaging faculty from different health professions programs, and 73.4% formally assed CP competencies of clinicians.

Table 5 displays an example of descriptive results using four variables collected from the network user survey—three predictor variables and one outcome variable. This analysis revealed that almost half (49.8%) often or routinely thought IPECP was essential in the process of patient care while 33.2% thought it never was. Also revealed from this descriptive analysis was that 64.9% of respondents had received instruction on team competencies and 53.6% had been exposed to IPE.

Table 6 displays bivariate results using the variable Team Care Provided as the outcome or dependent variable and the three predictor variables of IPECP is essential in the process of care, exposed to IPE, and instructed on team competencies. With alpha set at 0.05, all of the relationships tested were statistically significant. This bivariate analysis revealed that for respondents indicating that team care was provided (% Yes), 52.4% believed/thought that IPECP was often/routinely essential in the process of care. When team care was not provided (% No), 59.1% believed/thought that IPECP was never essential in the process of care. Likewise, for respondents indicating that team care was provided (% Yes), 67.7% had received instruction of team competencies; while of those respondents indicating that team care was not provided (% No), 59.1% indicated that they had not received instruction of team competencies. Finally, for respondents indicating that team care was provided (% Yes), 56.6% indicated that they had been exposed to IPE; while of those indicating that team care was not provided (% No), 72.7% indicated that they had not been exposed to IPE.

**Table 4:** Preliminary descriptive analysis of education survey NCDR data (linked to network user survey responders).

Variables and Factors		Count	Percent
Does your institution have a designated IPE Center/Office?	No	31	15.3
	Yes	171	84.2
	In Development	1	0.5
At your institution is there formal shared governance model (Co-leadership collaborative decision making) across the health professions schools regarding interprofessional education collaborative practice?	No	31	15.3
	Yes	172	84.7
Does your institution explicitly involve the leadership & administration of your major clinical partners in your IPE curriculum oversight planning and governance?	No	32	15.8
	Yes	171	84.2
At your institution does each major clinical site have an IPE champion or lead?	No	6	3.0
	Yes	197	97.0
At your institution does each major clinical site have a designated staff person to coordinate IPE?	No	38	18.7
	Yes	165	81.3
Do you have a formal method of engaging faculty from different health professions programs?	No	31	15.3
	Yes	172	84.7
At your institution do you formally assess CP competencies of your clinicians?	No	54	26.6
	Yes	149	73.4
At your institution have you developed IPE performance expectations for students?	No	51	25.1
	Yes	152	74.9
At your institution are the number of IPE experiences tracked over time for each student?	No	35	17.2
	Yes	168	82.8
At your institution do you have a formal IPE faculty development program?	No	51	25.1
	Yes	152	74.9

**Table 5:** Preliminary descriptive statistics for network user survey NCDR data.

Variable Type	Variables and Factors		Count	Percent
Predictor or Independent Variables	IPECP * Essential In Process of Care	Never	70	33.2
		Occasionally	36	17.1
		Often/Routinely	105	49.8
	Instructed on Team Competencies	No	74	35.1
		Yes	137	64.9
	Exposed to IPE **	No	98	46.4
		Yes	113	53.6
Outcome or Dependent Variables	Team Care Provided	No	22	10.4
		Yes	189	89.6

* Interprofessional Education and Collaborative Practice; ** Interprofessional Education.

**Table 6:** Preliminary bivariate analysis with team care provided as outcome or dependent variable.

Predictor Variables or Covariates		Team Care Provided		2-Sided Chi-Square *p* Values (alpha = 0.05)
		% No	% Yes	
IPECP Essential In Process Of Care	Never	59.1	30.2	0.022
	Occasionally	13.6	17.5	
	Often/Routinely	27.3	52.4	
Instructed on Team Competencies	No	59.1	32.3	0.013
	Yes	40.9	67.7	
Exposed to IPE	No	72.7	43.4	0.009
	Yes	27.3	56.6	

Table 7 displays the results of the multivariate analysis using the same predictor variables from the bivariate analysis and the dependent variable Team Care Provided. This analysis yielded that when team care was provided the clinicians or educators (responding to the network user survey) at the clinic performance sites had greater odds of believing/thinking that IPECP was essential in the process of care (Adjusted Odds Ratio 3.89, 95% CI 1.72–8.69). This effect size (adjusted odds ratio) indicates that clinicians or educators at clinics where team care was provided had 3.9 times higher odds of believing/thinking that IPECP was essential in the process of care.

**Table 7:** Preliminary logistic regression using team care provided as dependent variable.

Variables and Factors		Frequency	Adjusted Odds Ratio (95% CI)
IPECP Essential In Process of Care	Never	70	--*
	Occasionally	36	0.857 (0.358, 2.049)
	Often/Routinely	105	3.891 (1.742, 8.692)
Exposed to IPE	No	98	--*
	Yes	113	1.824 (0.934, 3.559)
Instructed on Team Competencies	No	74	--*
	Yes	137	1.089 (0.520, 2.278)

* Reference Category.

Table 8 displays the thematic analysis results of qualitative (or open text) data generated in response to the question: How is the patient and family considered in care plans? A high proportion of overall respondents to the network user survey (80.1%) answered this question. A content analysis of these data identified six distinct themes. These themes were:

Theme 1:  Patients participate in the development of care plans.
Theme 2:  The cost of care and other social determinant issues are taken into account as these relate to patients and their families.
Theme 3:  Asking the patient what they need.
Theme 4:  Patient and family are part of the care team.
Theme 5:  Patients and families are considered “all of the time”
Theme 6:  Do not know/uncertain of what the question is asking.

Examples of each of these themes are presented in Table 8. While only 171 responses are represented here, these data are illustrative of at the very least four things. First, open text fields can yield meaningful, analyzable data. Second, these qualitative data, once analyzed can and do result in information. Third, the information can be transformed into patterned, actionable knowledge. Fourth, over time and with sufficient responses, the results will allow for the identification of responses that can be used to develop response categories to non-open text questions.

**Table 8:** Six qualitative themes emergent from network user survey data question: how is the patient and family considered in care plans?

Themes	Examples of Responses Illustrating Themes
Patients participate in the development of care plans	A: As a primary care medical home we consider the patient at the center of the treatment plans. We include them in creating treatment plans by utilizing motivational interviewing and self-management tools. We use teach back and health literacy techniques to make sure patients understand the direction of their treatment. We print and go over the care plans at the end of each patient visit.
	B: They are the most vital component. If they don’t come I can’t do what I am trained to do. If they are not able to afford a medication I can write all the scripts I want but it won’t help. If they are not willing to make changes I cannot help.
The cost of care and other social determinant issues are taken into account as these relate to patients and their families.	A: Ability to afford therapies and transportation to clinic.
	B: I take into consideration social determinants of health when creating a plan.
	C: Social stressors must be addressed or care plan is useless.
Asking the patient what they need.	A: Ask questions such as: does the patient need a caretaker?
	B: Ask them what they want to do.
	C: Elicit patient goals and medication experiences (including those related to preferences, attitudes, beliefs, concerns, expectation and medication taking behavior) and use the answers to devise a pharmacotherapeutic plan with patient.
Patient and family are part of the care team.	A: Encourage family involvement to support positive change. Family is part of the treatment team.
	B: From start to finish engaging the patient in the team huddles and discussing the patient’s concerns. The patient/family are core in the structure and flow of the visits.
	C: Patient and family are part of the team and actions cannot proceed without their involvement.
Patients and families are considered “all of the time”	A: I would say almost always.
	B: Patient centered care.
	C: Patient/family centered care is our goal.
Don’t know/uncertain of what the question is asking.	A: I do not understand this question.
	B: What do you mean by “how do you consider…?”
	C: don’t know.

## 6. Conclusions

This paper has focused on describing and providing proof of concept for one of three intertwined knowledge generation strategies of the National Center on Interprofessional Practice and Education—the development of a sustainable informatics platform to complement and support the research agenda [13] and the NIN of IPECP interventions. These strategies, as they are implemented over time, will result in the production of empirically grounded knowledge regarding the direction and scope of the impact, if any, of IPECP on well-defined health and healthcare outcomes derived from the triple aim. Among the motivating factors for the National Center is the need for rigorously produced and scientifically sound evidence regarding IPECP. The deliberate and thoughtful development of a research agenda defined focused research questions to be examined [13]; the creation of a nexus innovation network developed the intervention research infrastructure needed to implement and test well-designed IPECP models linked to triple aim outcomes [14]; and the informatics platform described in this paper provides the data collecting, storing, and analyses foundation for knowledge generation. The work of the National Center to empirically ground, through extensive data collection and analyses, the potential impact of IPE on CP and eventually IPECP on health and healthcare outcomes has been a long time in coming to a field that has been in existence for forty-some odd years. The work is labor and resource intensive and, whatever the results, they will lead to additional research questions as established ones are answered. If we think of wisdom as integrated and generalizable knowledge made super-useful, then the analytic work facilitated by the informatics platform of the National Center will lead to the wisdom necessary to move the field of IPECP forward.
